# Supplementary material for: Regain flood adaptation in rice through a 14-3-3 protein OsGF14h
Source: Nat Commun. 2022 Sep 29;13:5664. doi: 10.1038/s41467-022-33320-x (PMC9522936; doi:10.1038/s41467-022-33320-x)
Supplement: Supplementary file 1 — Supplementary Information [file 41467_2022_33320_MOESM1_ESM.pdf]

Supplementary Information

Regain flood adaptation in rice through a 14-3-3 protein OsGF14h

Sun *et al*.

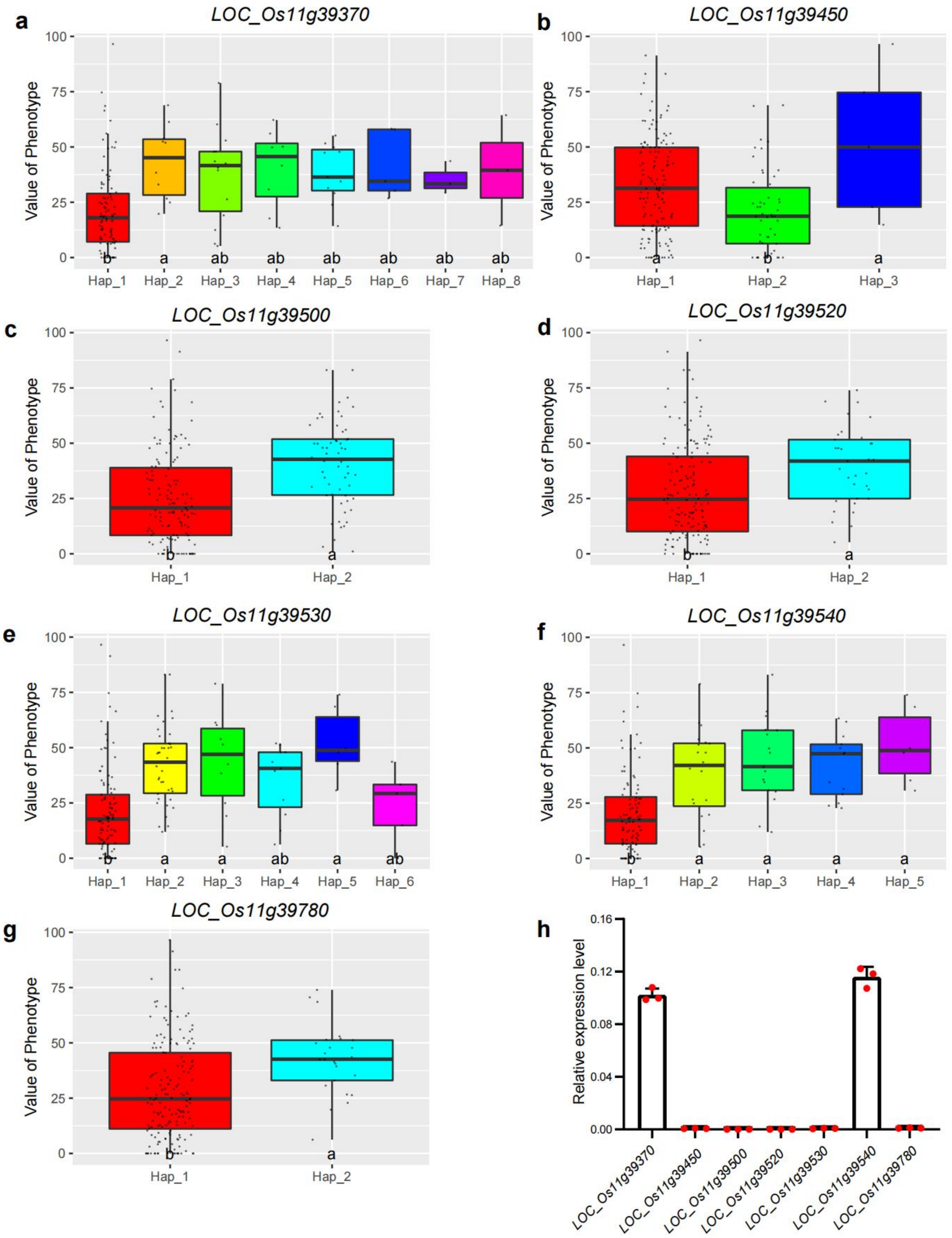

Supplementary Fig. 1 ***LOC_Os11g39540*** **(*OsGF14h*) is considered as the target candidate gene. (a, b, c, d, e, f, g)** Box plots for haplotype-based association analysis of the seven candidate genes. Dots represent the accessions, *n*=190 biologically independent samples. Center lines show the medians, box limits indicate the 25th and 75th percentiles, whiskers extend to 5th and 95th percentiles, as determined by CandiHap software software. **h** *LOC_Os11g39540* (*OsGF14h*) showed the highest expression level among seven candidate genes in the seeds of WR04-6 after 72 hour anaerobic treatment. Data are presented as means ± SD. *n* = 3 biologically independent samples. Source data are provided as a Source Data file.

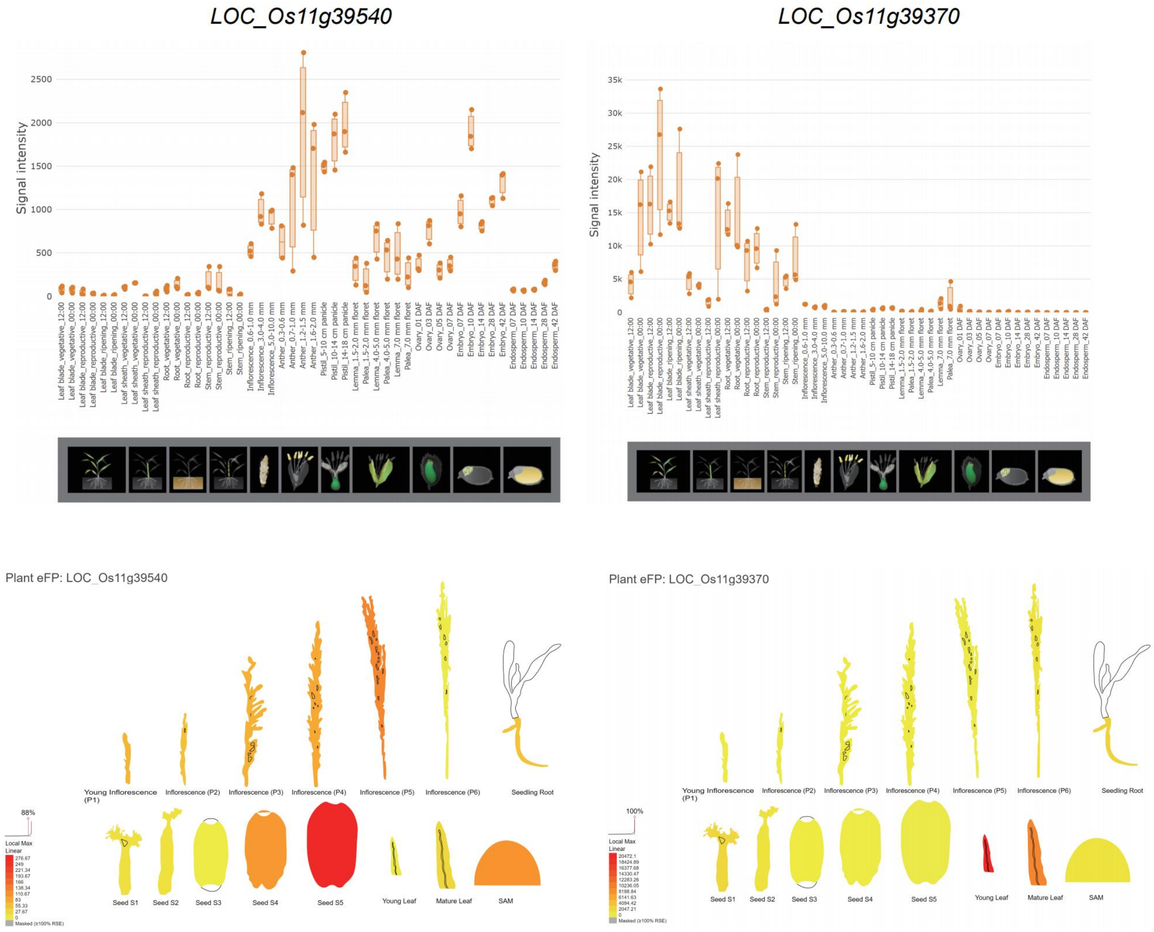

Supplementary Fig. 2 The spatio-temporal expression of *LOC_Os11g39540* and *LOC_Os11g39370* in Nipponbare based on RAP-DB and BAR database. The organs and tissues including leaf blade, leaf sheath, root, stem, inflorescence, anther, pistil, lemma, palea, ovary, embryo, endosperm. DAF, days after fertilization. RAP-DB, rice annotation project database; BAR, bio-analytic resource for plant biology. The graphs were wrote with downloaded the data and the Java script in the browser of local computer.

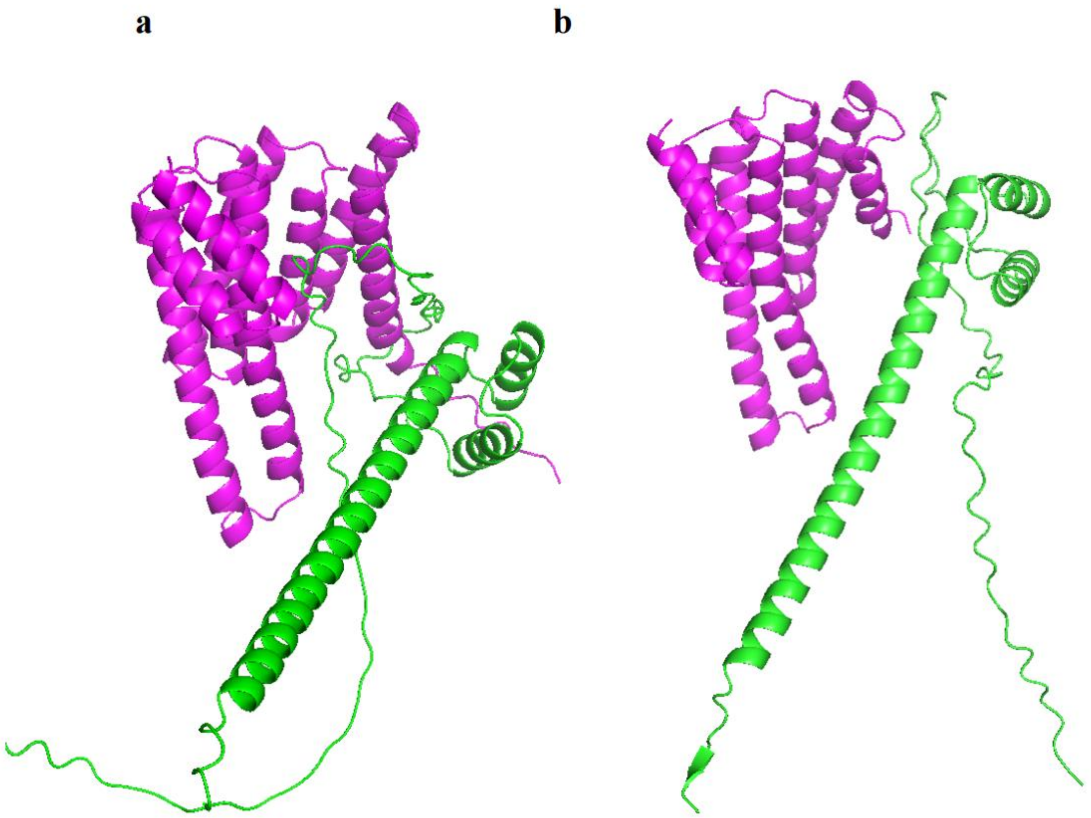

Supplementary Fig. 3 The secondary structure of the interaction between OsGF14h and OsHOX3 predicted by AlphaFold2. a The structure of the interaction between OsGF14h^{WR04-6} and OsHOX3. **b** The structure of the interaction between OsGF14h^{SN9816} and OsHOX3.

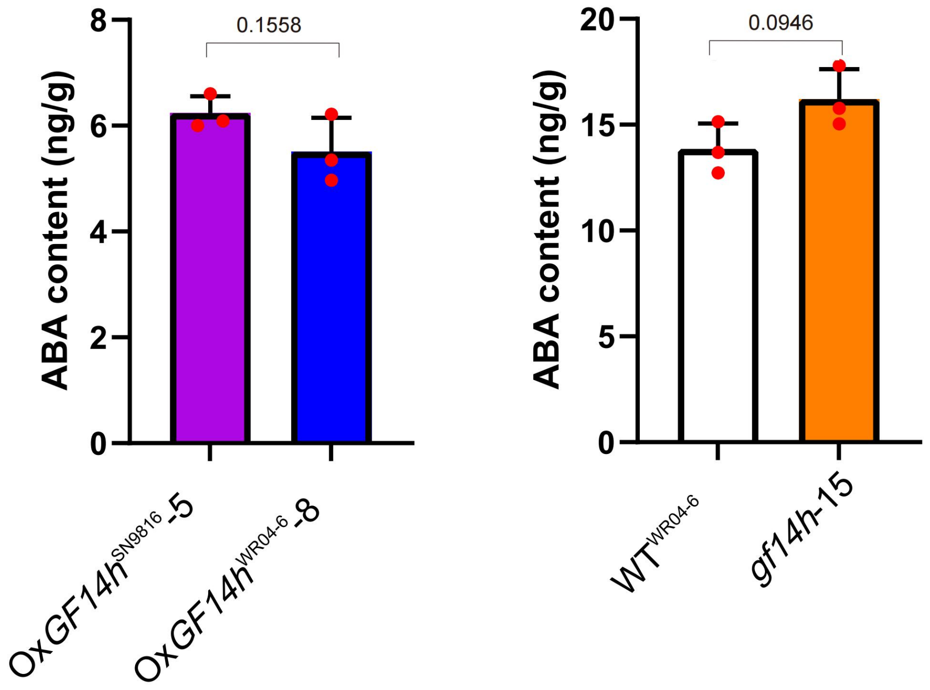

Supplementary Fig. 4 The content of endogenous ABA between overexpression lines Ox*OsGF14h*^{SN9816}-5 and Ox*OsGF14h*$^{WR04-6}$-8 , and WT^{WR04-6} and knockout line *gf14h*-15. Data are presented as means ± SD. *n* = 3 biologically independent samples. *P*-values are indicated by unpaired two-tailed Student's *t*-test. Source data are provided as a Source Data file.

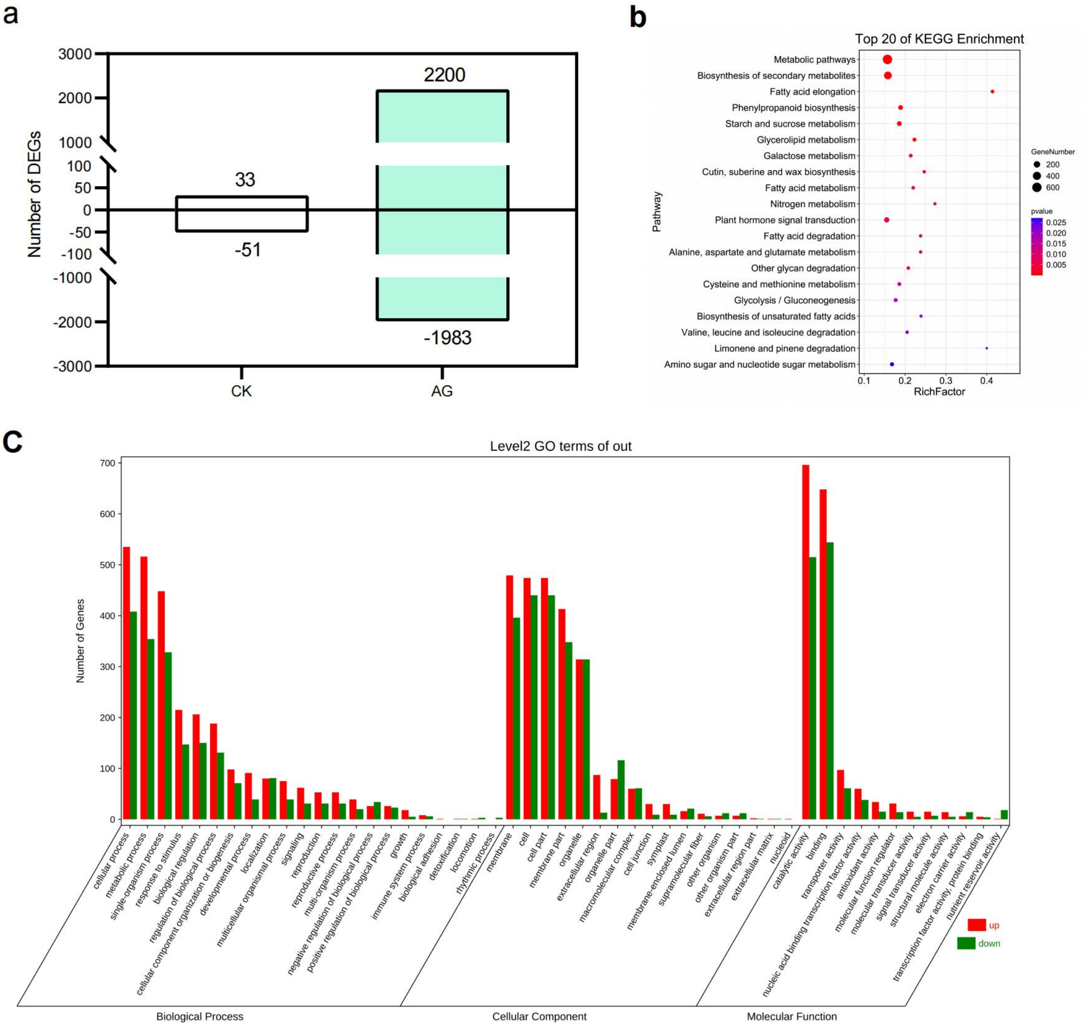

Supplementary Fig. 5 Enrichment and relative expression analysis of DEGs for Ox*OsGF14h*$^{\mathrm{WR04\text{-}6}}$-8 and Ox*OsGF14h*$^{\mathrm{SN9816}}$-5 based on RNA sequencing under anaerobic (AG) and aerobic (CK) conditions. a Numbers of DEGs of Ox*OsGF14h*$^{\mathrm{WR04\text{-}6}}$-8 and Ox*OsGF14h*$^{\mathrm{SN9816}}$-5 seeds under AG and CK conditions at the 12th hour according to RNA sequencing. **b** GO enrichment anaylsis of DEGs under AG condition. **c** Top 20 of KEGG enrichment analysis of DEGs under AG condition. DEGs, differential expression genes; GO, Gene Ontology; KEGG, Kyoto Encyclopedia of Genes and Genomes. Source data are provided as a Source Data file.

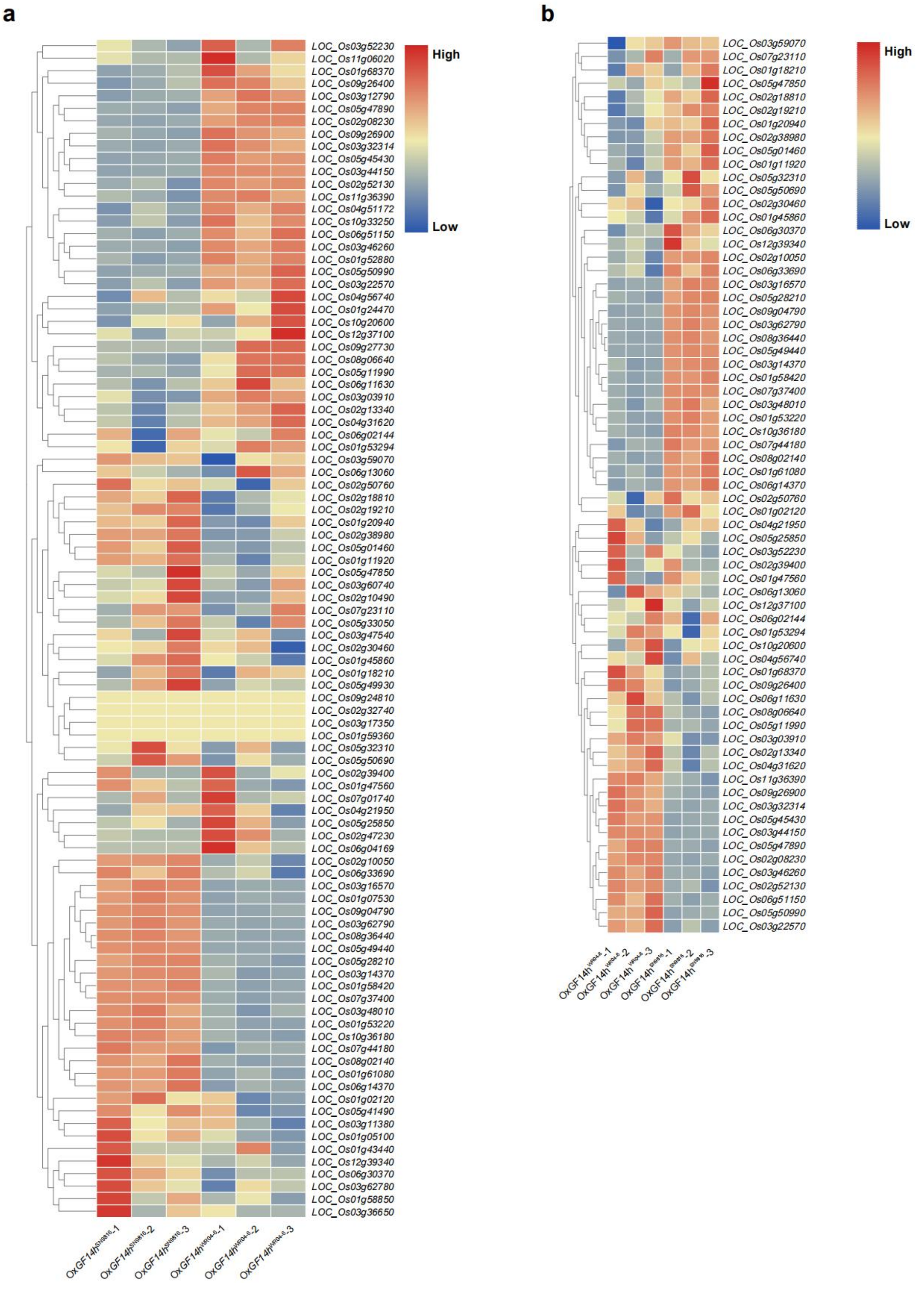

Supplementary Fig. 6 Heatmaps of relative expression of ABA signaling pathway genes of Ox*OsGF14h*$^{WR04-6}$ and Ox*OsGF14h*^{SN9816} under anaerobic conditions at the 12th hour. **a** ABA-responsive genes (GO:0009738). **b** ABA signaling-related genes (GO:0009737). The colour key (blue to red) represents the expression level from low to high. *n* = 3 biologically independent samples. Source data are provided as a Source Data file.

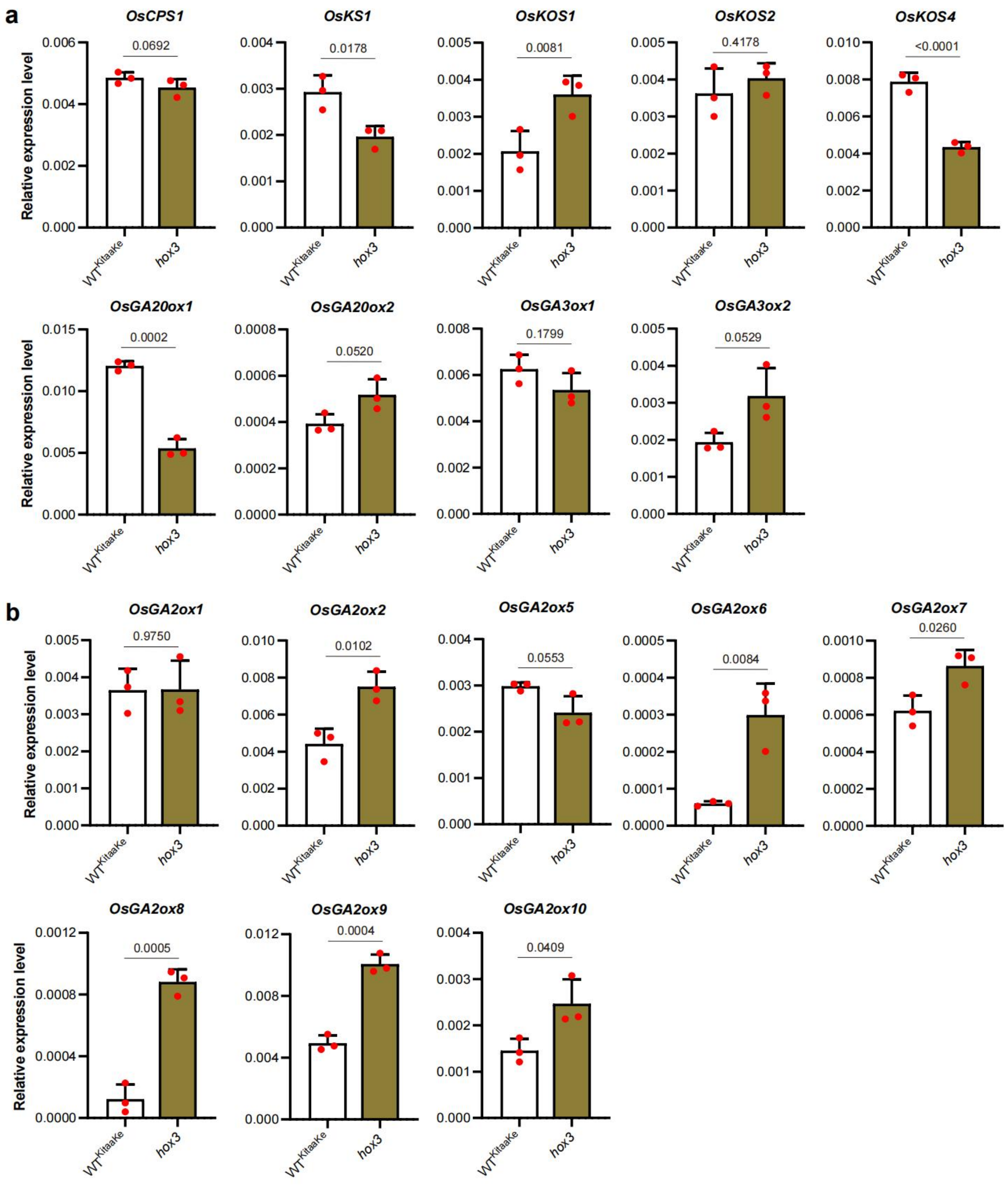

Supplementary Fig. 7 Relative expression level of key genes in GA biosynthesis pathway of WT^{kitaake} and *hox3* mutant under anaerobic condition. a Relative expression level of genes in the GA biosynthesis pathway. **b** Relative expression level of genes in the GA degradation pathway. Data are presented as means ± SD. *n* = 3 biologically independent samples. *P*-values are indicated by unpaired two-tailed Student's *t*-test. Source data are provided as a Source Data file.

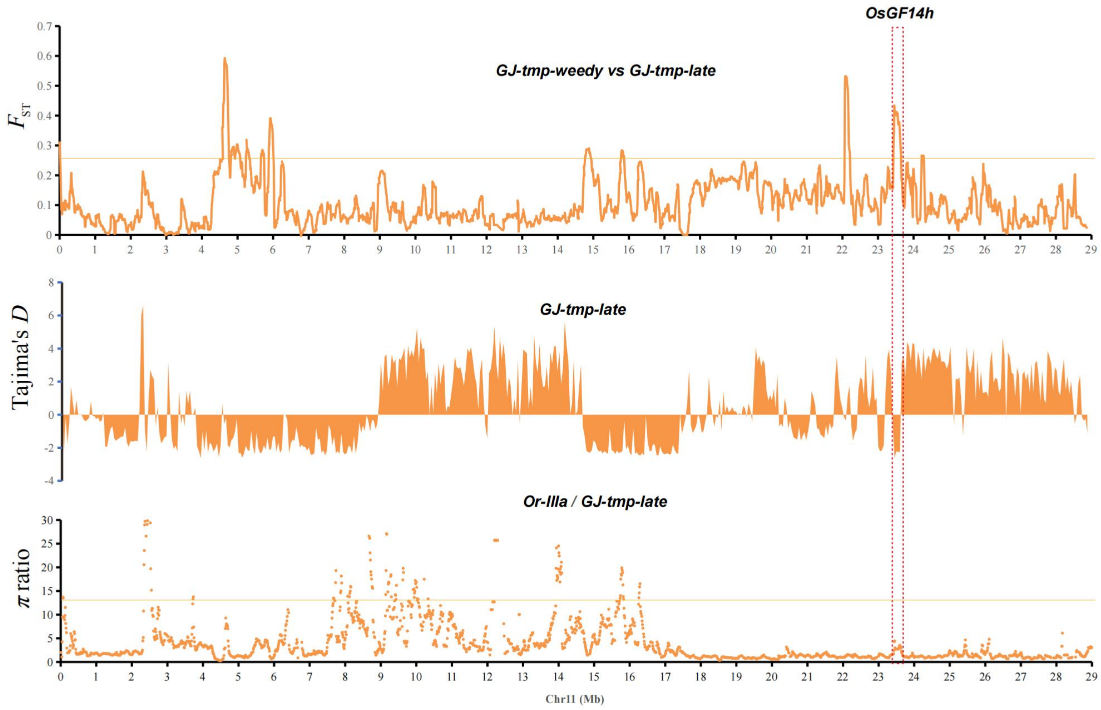

Supplementary Fig. 8 Population differentiation and selection analysis for *GJ-tmp-late* and *GJ-tmp-weedy* on chromosome 11. Population differentiation analysis between *GJ-tmp-late* and *GJ-tmp-weedy*. The *y* axis on top spectrum represents 100kb window-based F_{ST} values, the horizontal solid line corresponds to a 0.05 significance level of F_{ST} values. The *y* axis on the middle spectrum represents 500kb window-based Tajima' *D* values of *GJ-tmp-late*. Selection sweep is defined by *π* ratio (*πOr-IIIa / πGJ-tmp-late*). The *y* axis on the bottom spectrum represents the *π* ratio, horizontal solid line corresponding to 0.05 significance level of threshold. The *x* axis of the three spectrums represent physical position of chromosome 11. *OsGF14h* genomic region is marked with vertical dashed boxes. This source data shares the same set with Fig. 6b that are provided as a Source Data file.

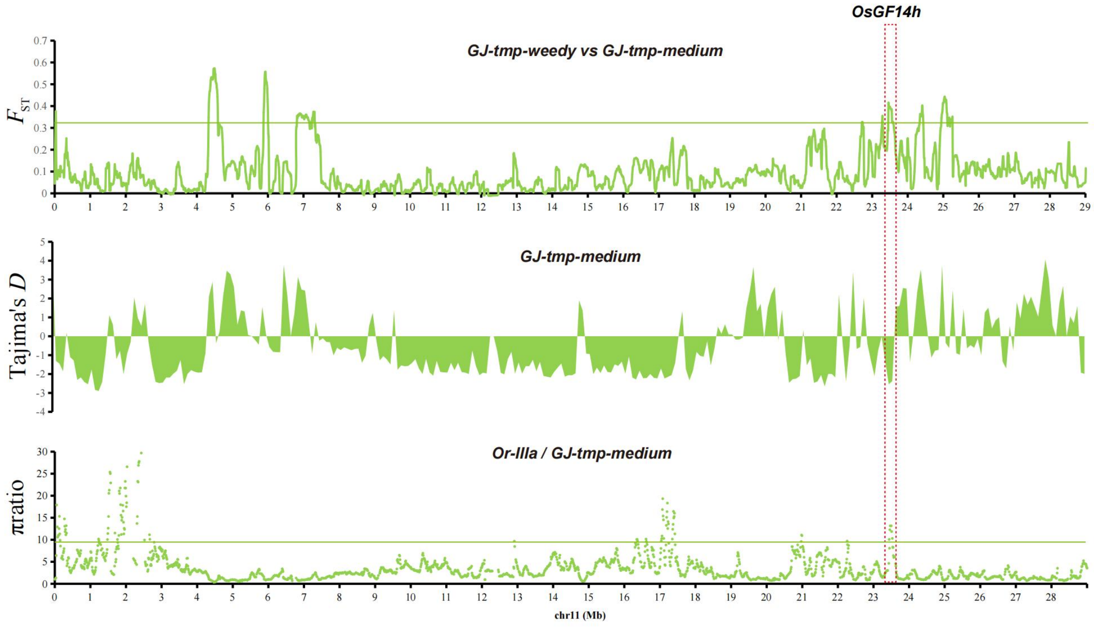

Supplementary Fig. 9. Population differentiation and selection analysis for *GJ-tmp-medium* and *GJ-tmp-weedy* on chromosome 11. Population differentiation analysis between *GJ-tmp-medium* and *GJ-tmp-weedy*. The *y* axis on top spectrum represents 100kb window-based F_{ST} values, the horizontal solid line corresponds to a 0.05 significance level of F_{ST} values. The *y* axis on the middle spectrum represents 500kb window-based Tajima' *D* values of *GJ-tmp-medium*. Selection sweep is defined by *π* ratio (*πOr-IIIa* / *πGJ-tmp-medium*). The *y* axis on the bottom spectrum represents the *π* ratio, horizontal solid line corresponding to 0.05 significance level of threshold. The *x* axis of the three spectrums represent physical position of chromosome 11. *OsGF14h* genomic region is marked with vertical dashed boxes. This source data shares the same set with Fig. 6c that are provided as a Source Data file.

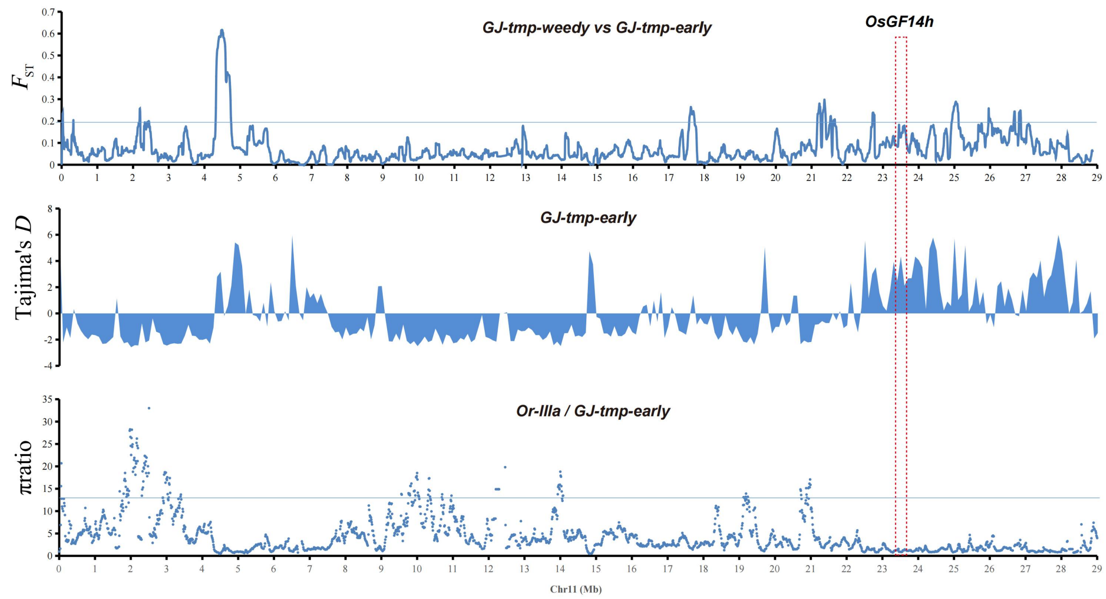

Supplementary Fig. 10. Population differentiation and selection analysis for *GJ-tmp-early* and *GJ-tmp-weedy* on chromosome 11. Population differentiation analysis between *GJ-tmp-early* and *GJ-tmp-weedy*. The *y* axis on top spectrum represents 100kb window-based F_{ST} values, the horizontal solid line corresponds to a 0.05 significance level of F_{ST} values. The *y* axis on the middle spectrum represents 500kb window-based Tajima' *D* values of *GJ-tmp-early*. Selection sweep is defined by π ratio (π*Or-IIIa* / π*GJ-tmp-early*). The *y* axis on the bottom spectrum represents the π ratio, horizontal solid line corresponding to 0.05 significance level of threshold. The *x* axis of the three spectrums represent physical position of chromosome 11. *OsGF14h* genomic region is marked with vertical dashed boxes. This source data shares the same set with Fig. 6c that are provided as a Source Data file.

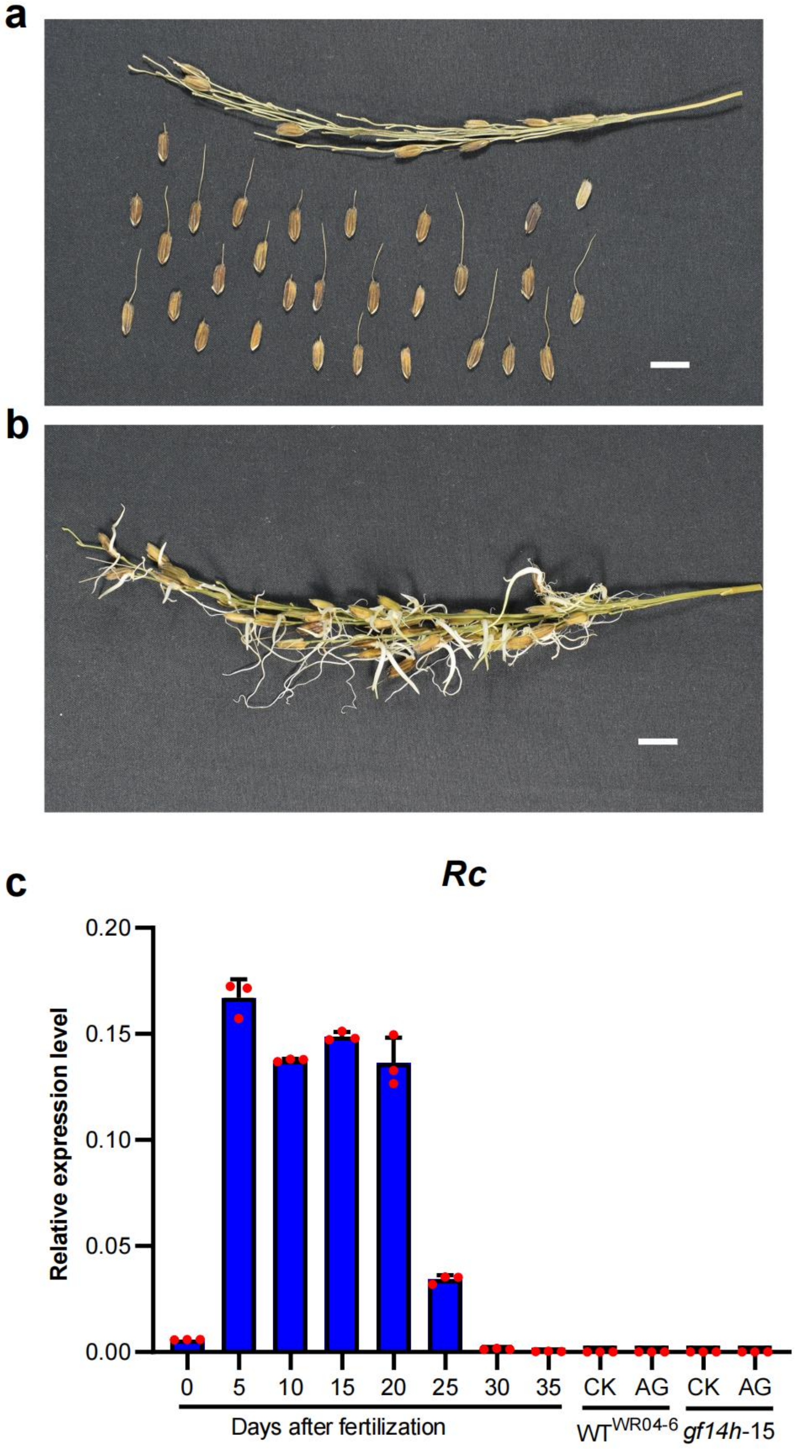

Supplementary Fig. 11. *Rc* reduce pre-harvest sprouting. The performance of pre-harvest sprouting (PHS) of WT^{WR04-6} **(a)** and *Rc* knock out line *rc*-6 **(b)** during the seed maturation process. Bar = 1 cm. **c** Relative expression level of *Rc* during the seed maturation process of WT^{WR04-6}, and on the 4th day of aerobic (CK) and anaerobic germination (AG) of WT^{WR04-6} and knockout line *gf14h-15*. Data are presented as mean ± SD, n = 3 biologically independent samples. Source data are provided as a Source Data file.

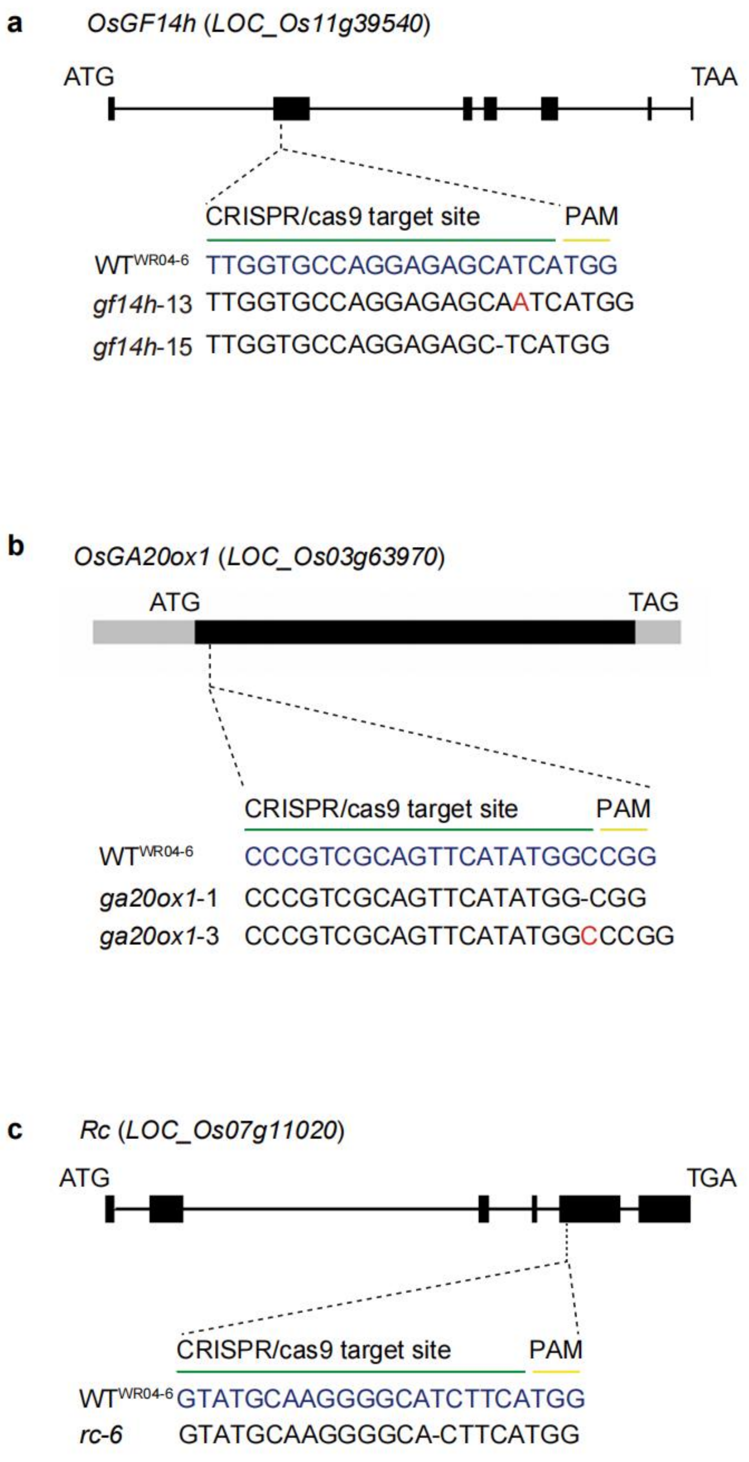

Supplementary Fig. 12. The mutation positions and types of CRISPR/Cas9 knockout lines, *OsGF14h* (a)*, OsGA20ox1* (b), *and Rc* (c). The black square represent the exons of genes. WT, wild type. The green lines indicate CRISPR/Cas9 target sites and yellow line indicate PAM sequence.
