# Supplementary material for: Regain flood adaptation in rice through a 14-3-3 protein OsGF14h
Source: Nat Commun. 2022 Sep 29;13:5664. doi: 10.1038/s41467-022-33320-x (PMC9522936; doi:10.1038/s41467-022-33320-x)
Supplement: Supplementary file 3 — Description of Additional Supplementary Files [file 41467_2022_33320_MOESM3_ESM.pdf]

Description of Additional Supplementary Files

File Name: Supplementary Data 1.
Description: Phenotype of anaerobic germination of recombinant inbred lines and parents.

File Name: Supplementary Data 2.
Description: Sample germination rates in GWAS panel after 72 hours of anaerobic treatment.

File Name: Supplementary Data 3.
Description: The annotation of 7 candidate genes.

File Name: Supplementary Data 4.
Description: Raw data for the response to the second question of reviewer #4 in Point-by-point response-version 2.

File Name: Supplementary Data 5.
Description: Statistics of haplotype types among 1596 rice accessions from nine ecotypes.

File Name: Supplementary Data 6.
Description: Haplotype frequency of *OsGF14h*^{Hap1} in each ecotype.

File Name: Supplementary Data 7.
Description: Haplotypes of *OsGF14h* and *Sdr4* among 1596 rice accessions from nine ecotypes.

File Name: Supplementary Data 8.
Description: Primer and probe sequences used in this study.
